# Organ Length Control by an ADAMTS Extracellular Protease in *Caenorhabditis elegans*

**DOI:** 10.1534/g3.116.028019

**Published:** 2016-03-17

**Authors:** Yukimasa Shibata, Yuri Kawakado, Noriyoshi Hori, Kota Tanaka, Ryo Inoue, Tomomi Takano, Yukihiko Kubota, Kiyoji Nishiwaki

**Affiliations:** * Department of Bioscience, Kwansei Gakuin University, 2-1 Gakuen, Sanda 669-1337, Japan; † Department of Developmental Biology and Neurosciences, Graduate School of Life Sciences, Tohoku University, 2-1-1 Katahira, Aoba-ku, Sendai 980-8577, Japan

**Keywords:** organ elongation, ADAMTS protease, basement membrane, pharynx

## Abstract

MIG-17, a secreted protease of the ADAMTS family, acts in the directed migration of gonadal distal tip cells (DTCs) through regulation of the gonadal basement membrane in *Caenorhabditis elegans*. Here, we show that MIG-17 is also required for the control of pharynx elongation during animal growth. We found that the pharynx was elongated in *mig-17* mutants compared with wild type. MIG-17 localized to the pharyngeal basement membrane as well as to the gonadal basement membrane. The number of nuclei in the pharynx, and the pumping rate of the pharynx, were not affected in *mig-17* mutants, suggesting that cells constituting the pharynx are elongated, although the pharynx functions normally in these mutants. In contrast to the control of DTC migration, MIG-18, a secreted cofactor of MIG-17, was not essential for pharynx length regulation. In addition, the downstream pathways of MIG-17 involving LET-2/type IV collagen, FBL-1/fibulin-1, and NID-1/nidogen, partly diverged from those in gonad development. These results indicate that basement membrane remodeling is important for organ length regulation, and suggest that MIG-17/ADAMTS functions in similar but distinct molecular machineries in pharyngeal and gonadal basement membranes.

Organogenesis in animal development often involves elongation of tissues. For example, convergent extension tissue movements elongate the anterior-posterior axis of the vertebrate body plan (Keller 2002). Elongation of tubular epithelia occurs by cell shape change, cell rearrangement, and cell proliferation in both vertebrates and invertebrates (Andrew and Ewald 2010). While intracellular mechanisms have been the major focus of these studies, extracellular molecular mechanisms operating in organ elongation remain poorly understood. In the present study, we showed that a secreted metalloprotease of the ADAMTS (a disintegrin and metalloprotease with thrombospondin motifs) family acts in organ length regulation through modulating the basement membrane in *C. elegans*.

ADAMTS family proteins have received considerable attention because defects in many of these proteins have been linked to hereditary diseases related to disorders affecting the extracellular matrix (ECM) (Dubail and Apte 2015; Hubmacher and Apte 2015). We have been studying the function of the *C. elegans mig-17* gene, which encodes an ADAMTS required for the regulated migration of the gonadal leader cells called distal tip cells (DTCs) (Kim and Nishiwaki 2015). MIG-17 is secreted from body wall muscle cells, and localizes to the basement membrane of the developing gonad, and acts in the directed migration of DTCs to generate the U-shaped gonad arms (Nishiwaki *et al.* 2000).

Genetic suppressor analyses of *mig-17* mutants identified dominant gain-of-function (*gf*) mutations in two genes that encode basement membrane proteins, FBL-1/fibulin-1, and LET-2/α2 subunit of collagen IV (Kubota *et al.* 2004, 2008). The suppressor *fbl-1(gf)* mutations consist of substitutions of evolutionarily conserved amino acids within the second EGF-like motif of FBL-1C and D isoforms. Of these, the mutant FBL-1C protein acts in the suppression of *mig-17* DTC migration defects. FBL-1C is secreted from the intestine, and recruited to the gonadal basement membrane by MIG-17 activity, where it is likely to be activated by MIG-17, and functions in directional control of DTC migration (Kubota *et al.* 2004). The suppression by *fbl-1(gf)* mutations depends on NID-1/nidogen, a basement membrane protein (Kubota *et al.* 2008). The two suppressor *let-2(gf)* mutations result in amino acid changes in the triple helical domain, and in the C-terminal noncollagenous domain. LET-2 protein that is localized to the gonadal basement membrane can be activated (cleaved or conformationally changed) by MIG-17 to control DTC migration. In contrast to *fbl-1(gf)* mutations, suppression by *let-2(gf)* mutations is NID-1 independent (Kubota *et al.* 2008). In addition to these basement membrane proteins, we recently identified a novel secreted protein, MIG-18, which acts as a cofactor of MIG-17 in the control of DTC migration (Kim *et al.* 2014). Like MIG-17, MIG-18 is secreted from the body wall muscle cells, and localizes to the gonadal basement membrane.

Here we report that *mig-17* functions in the control of pharynx length. The pharynx is a complex organ made up of 62 cells, which includes epithelial cells, muscle cells, marginal cells, grand cells, and neurons (Altun and Hall 2009). The pharynx is an epithelial organ surrounded by a basement membrane. It is ∼60 μm long at hatching and elongates during four larval stages to ∼130 μm at the young adult stage. Although pharynx length did not differ between wild-type and *mig-17* mutant animals during the first larval stage, the *mig-17* pharynx became longer than that of wild type until the adult stage. We showed that the function of MIG-17 was as important for the control of pharynx length as for its function in DTC migration. Genetic analyses of genes that act in the MIG-17 pathway to regulate DTC migration, most of which are basement membrane proteins, revealed shared and organ-specific aspects of the molecular mechanisms regulating DTC migration and pharynx length. These results suggest that, although MIG-17/ADAMTS acts in the remodeling of multiple basement membranes, it can function in distinct molecular contexts depending on the tissues and organs.

## Materials and Methods

### Strains and genetic analysis

Culture and handling of *C. elegans* were conducted as described (Brenner 1974). Worms were cultured at 20°. The following mutations were used in this work: *mig-17(k113*, *k135*, *k167*, *k169*, *k174)* (Nishiwaki 1999; Nishiwaki *et al.* 2000), *mig-18(k140*, *tm2007)* (Kim *et al.* 2014), *fbl-1(k201*, *k206*, *tk45)* (Kubota *et al.* 2004), *let-2(k193*, *k196)* (Kubota *et al.* 2008), *nid-1(cg118*, *cg119)* (Kang and Kramer 2000), *unc-42(e270)*, *unc-119(e2498)* (Brenner 1974; Maduro and Pilgrim 1995), *emb-9(b117*, *b189*, *g34)* (Gupta *et al.* 1997), *cle-1(cg120)* (Ackley *et al.* 2001), *sdn-1(ok449)* (Minniti *et al.* 2004), *mig-6(k177)* (Kawano *et al.* 2009), *mig-23(k180)* (Nishiwaki *et al.* 2004), *mig-22(k141)* and *sqv-5(k172*, *k175)* (Suzuki *et al.* 2006) and *cogc-1(k179)*, *cogc-3(k181)* (Kubota *et al.* 2006). The *mig-17(k160)* was isolated by ethyl methanesulfonate mutagenesis and failed to complement *mig-17(k174)*.

### Microscopy

Pharynx length, body length and gonad migration phenotypes were determined using a Nomarski microscope (Axioplan 2; Zeiss). Pharynx and body length were analyzed using Image J software. Pharynx length was assessed by measuring the length of the pharyngeal region excepting the buccal cavity. Pharynx volume was assessed by measuring the area of the sagittal optical section of the pharynx. Analysis of gonadal phenotypes was performed at the young adult stage as described (Nishiwaki 1999). The patterns of expression of Venus fusion proteins were analyzed using a confocal laser scanning microscope (LSM5) equipped with a Plan-Neofluar 40× lens and controlled by PASCAL version 3.2 SP2 software (all from Zeiss).

### Analysis of nuclear counts

Wild-type and *mig-17(k174)* young adult hermaphrodites with the transgenic markers *qIs147[sur-5*::*gfp]* (Kershner *et al.* 2014) and *tkTSi1[emb-9*::*mCherry]* were used. *tkTSi1[emb-9*::*mCherry]* was constructed as follows: the *Avr*II and *Spe*I restriction fragment from the *emb-9*::*mCherry* construct (Ihara *et al.* 2011) was inserted at the *Spe*I site of the miniMos vector pCFJ909, and the resulting plasmid was used to generate an animal having a single-copy insertion of *emb-9*::*mCherry* in linkage group I, *tkTSi1[emb-9*::*mCherry]* (Frokjaer-Jensen *et al.* 2014). The confocal images were obtained with a 0.5-μm *z*-series using a spinning-disk confocal scan head (CSU-X1; Yokogawa) that was mounted on a Zeiss Imager M2 microscope equipped with an EM-CCD camera (ImageEM; Hamamatsu Photonics). The nuclei surrounded by the pharyngeal basement membrane were counted.

### Analysis of amphid sensory neurons

DiI staining of amphid neurons was performed as described (Hedgecock *et al.* 1985). Nomarski and fluorescence microscopy was performed using a Zeiss Axioplan 2 microscope equipped with both optical systems.

### Data availability

The authors state that all data necessary for confirming the conclusions presented in the article are represented fully within the article.

## Results

### mig-17 is required to achieve a pharynx of normal length

**Figure 1:**
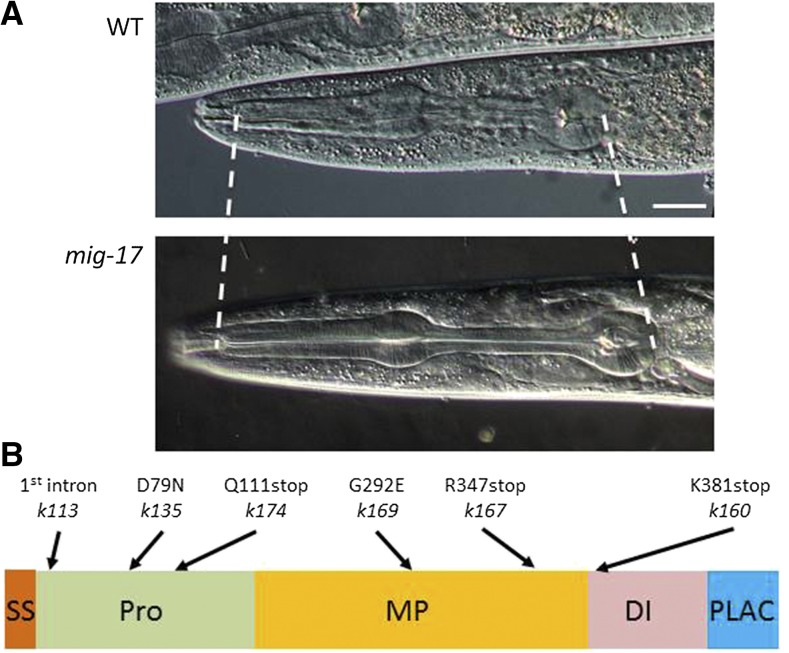
Pharynx elongation phenotype and mutation sites of *mig-17* mutants. (A) Nomarski micrographs of pharynges of wild-type and *mig-17(k174)* young adult hermaphrodites. Dashed lines depict anterior and posterior ends of the pharynx. Anterior is to the left, dorsal to the top. Bar: 20 μm. (B) *mig-17* mutation sites. Positions of mutations are indicated along with the domain structure of the MIG-17 protein. SS, signal sequence; Pro, prodomain; MP, metalloprotease domain; DI, disintegrin domain; PLAC, protease and lacunin domain.

**Figure 2:**
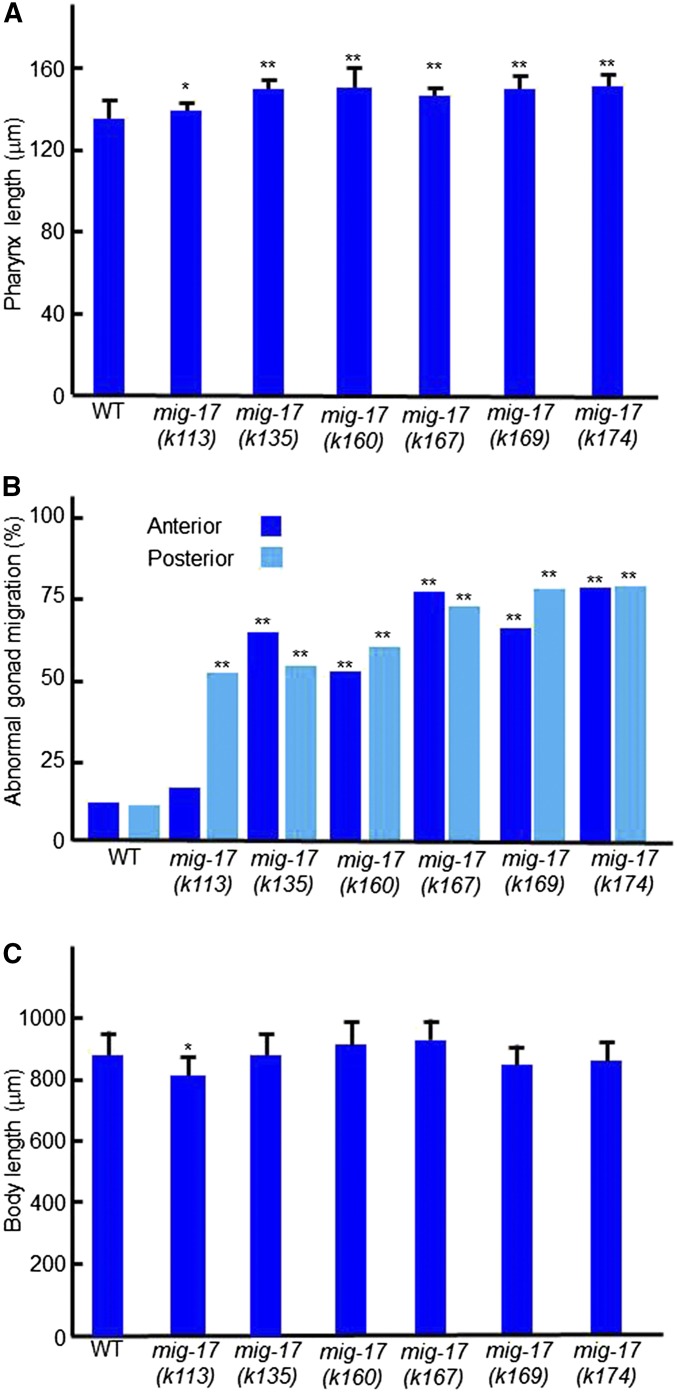
Pharyngeal and gonadal phenotypes of *mig-17* mutants. Young adult hermaphrodites were analyzed. (A–C) Pharynx lengths (*n* = 20) (A), % DTC migration defects (*n* = 60) (B), and body length (*n* = 20) (C) for each allele are shown. Data are shown as the mean ± SD for (A) and (C). *P*-values for Fisher’s exact test against wild type (WT) are indicated: ** *P* < 0.01, * *P* < 0.05.

*mig-17* was originally identified through mutations affecting directional migration of gonadal leader cells, the DTCs, in *C. elegans* (Nishiwaki *et al.* 2000). We found that the gene is also required to form a pharynx of the proper length: *mig-17* mutants exhibited an elongated pharynx phenotype (Figure 1A). We analyzed six mutant alleles of *mig-17*, *k113*, *k135*, *k160*, *k167*, *k169*, and *k174* (Figure 1B). We measured body length in addition to pharynx length to determine whether mutations affect pharyngeal length specifically or they affect overall size of the animal. All six *mig-17* mutants showed pharynges that were significantly longer than those in the wild type. The putative null allele, *k174*, which had the strongest effect on DTC migration, also exhibited the strongest phenotype with respect to pharynx length. The *k135* and *k160* alleles, which had moderate effects on DTC migration, exhibited strong pharyngeal phenotypes comparable to that in *k174* (Figure 2, A–C).

**Figure 3:**
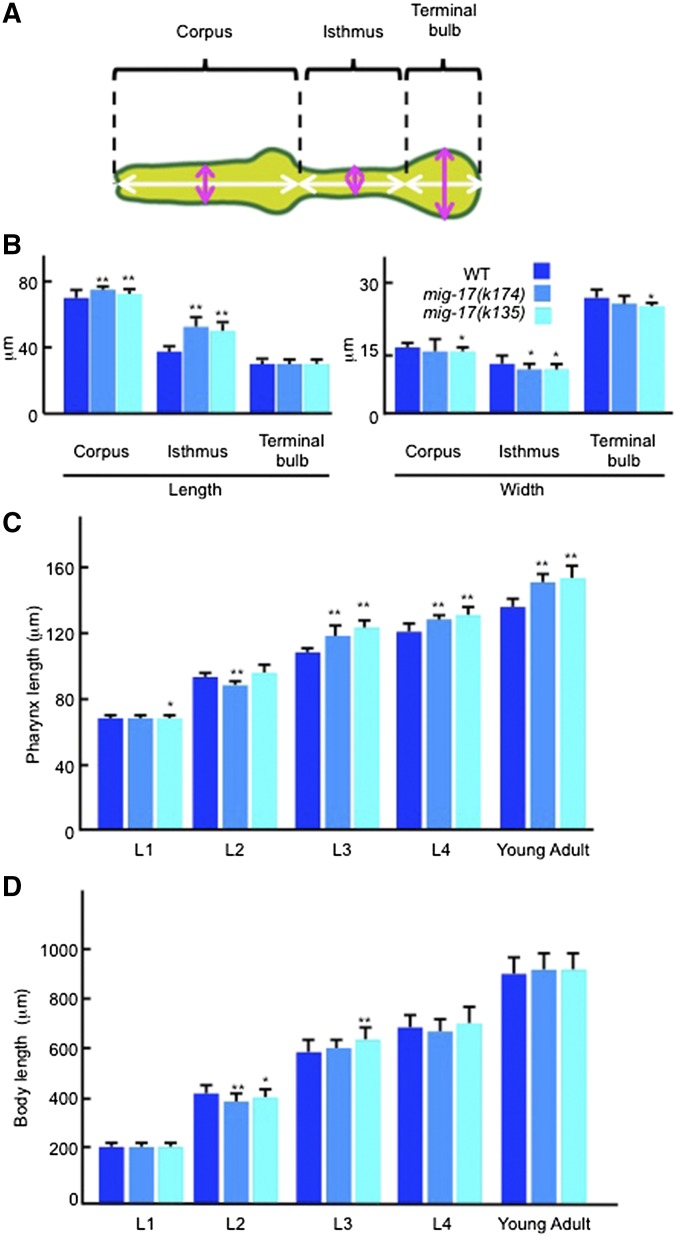
Pharyngeal defects in *mig-17* mutants. (A) The regions of the pharynx are indicated. Corpus, isthmus, and terminal bulb correspond to the muscular regions from pm1 to pm4, pm5, and from pm6 to pm8, respectively. Regions measured to determine length and width in (B) are shown by white and magenta bidirectional arrows, respectively. (B) Length and width of each pharyngeal region in young adult hermaphrodites (*n* = 20). The width was measured at the center of the corpus, isthmus, and terminal bulb. (C, D) Pharyngeal development in wild-type and *mig-17* mutant animals (*n* = 20). Pharynx length (C) and body length (D). Worms having ∼10 germline nuclei, having gonad arms that recently completed the first turn and having Christmas tree-shaped vulval primordia were used as L2, L3, and L4 stage animals, respectively. Data are shown as the mean ± SD. *P*-values for Fisher’s exact test against WT are indicated: ** *P* < 0.01, * *P* < 0.05.

The pharynx can be divided into three parts, the corpus, isthmus and terminal bulb, from anterior to posterior (Figure 3A). We examined which part is elongated in the *mig-17* mutants. In the strong *k174* and *k135* alleles, the corpus and isthmus were elongated, but the terminal bulb was not. In particular, lengthening of the isthmus was prominent in these alleles. As for the thickness of the pharyngeal tube, we detected slight thinning of the isthmus in both alleles relative to wild type (Figure 3B). We assessed pharynx volumes by examining the area of the sagittal optical section of wild type and mutant pharynges, and found no significant difference between them: 2354 ± 93 μm^2^ (*n* = 20) for wild type and 2377 ± 129 μm^2^ (*n* = 20) for *mig-17(k174)* (*P* = 0.528). These results suggest that the pharynx in *mig-17* mutants extends along the anterior-posterior axis, and decreases in width without affecting its volume.

To understand developmental stages when MIG-17 is required for normal pharynx length, we examined when the pharyngeal abnormality becomes evident in *k135* and *k174* mutant animals. In both these alleles, pharynx elongation was observed later than the third larval (L3) stage but not during the L2 stage. Thus, it is possible that *mig-17* function is required for pharynx length regulation after the L2 stage (Figure 3C).

### Nuclear numbers, pumping rates, and length of the amphid sensory dendrites are not affected in *mig-17* mutants

The difference in pharynx length between the wild type and mutants could reflect a difference either in cell number or cell length. We examined the number of nuclei in the pharynx in young adult animals expressing SUR-5-GFP and EMB-9-mCherry, markers for somatic nuclei and the basement membrane, respectively. The nuclear counts were at most 80; 79 ± 1.9 (*n* = 3) in the wild type, and 80 ± 0.4 (*n* = 5) in *mig-17(k174)*. The numbers of nuclei in pm5 and mc2 cells constituting the isthmus were six and three, respectively, in all *mig-17* and wild type animals examined, suggesting that nuclear number was not increased in the *mig-17* mutants. Thus, it is likely that the length of cells constituting the pharynx is affected in the mutants.

**Figure 4:**
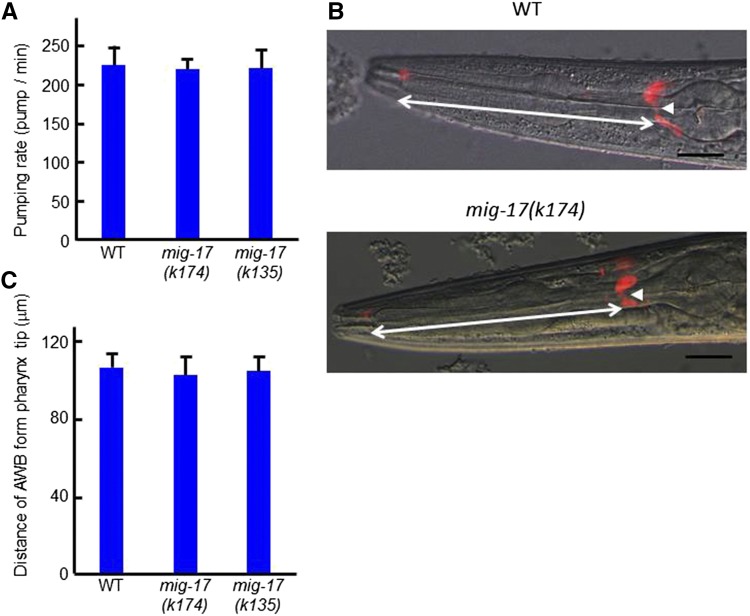
Pharyngeal pumping rates and length of AWB dendrites in *mig-17* mutants. Young adult hermaphrodites were analyzed. (A) Pumping rates of WT and *mig-17* animals. (B) Fluorescence and Nomarski merged images of WT and *mig-17* animals stained with DiI. The cell body of the AWB neuron is indicated by arrowheads. (C) Distances of AWB neuronal cell bodies from the pharyngeal tip, shown by bidirectional arrows in (B). Data are shown as the mean ± SD (*n* = 20).

Pumping frequencies did not differ between wild type and *mig-17(k174)* mutants (Figure 4A), suggesting that pharyngeal elongation does not affect pharyngeal function. We then examined whether *mig-17* mutants affect the length of amphid sensory dendrites surrounding the pharynx. Labeling of the sensory neurons with DiI revealed that they were positioned and extended their sensory dendrites normally (Figure 4, B and C). Thus, *mig-17* appears to control the length of the pharynx specifically.

### Catalytic activity, disintegrin (DI) domain, and N-glycosylation are essential for pharynx length regulation

**Figure 5:**
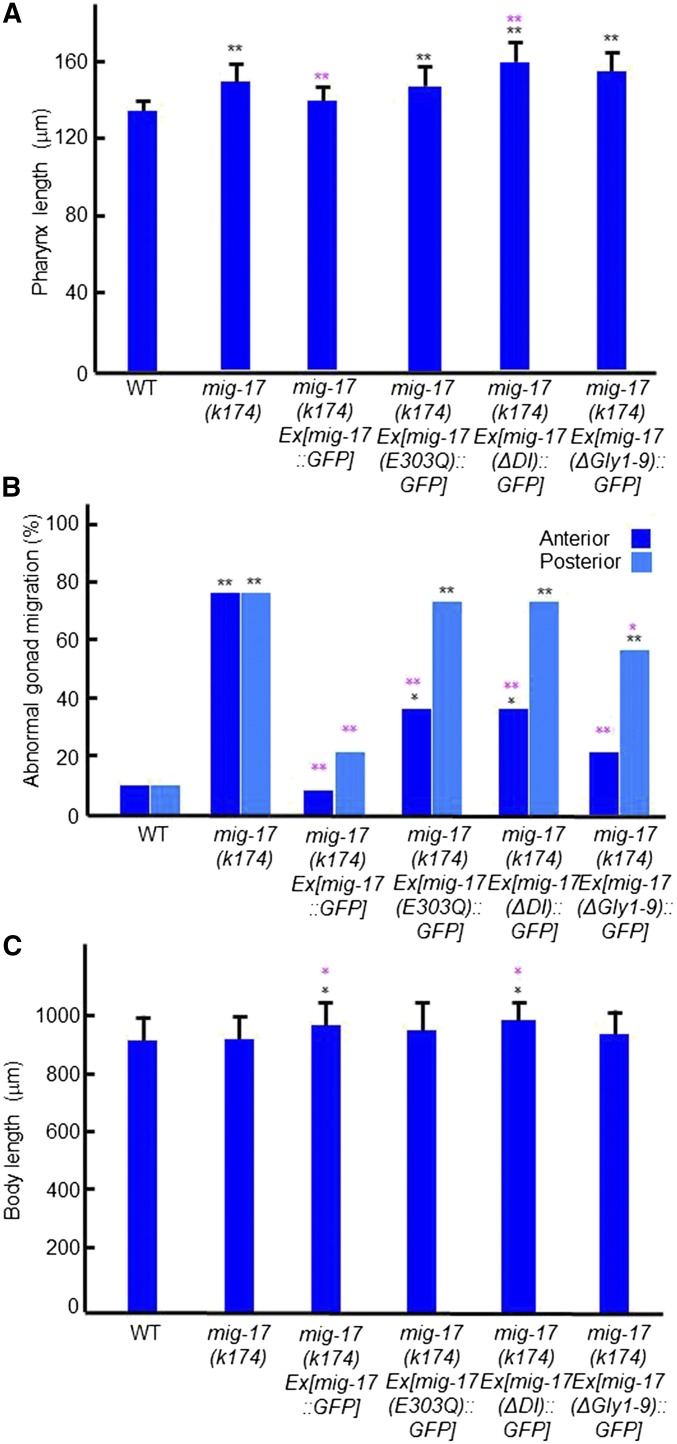
Transgenic rescue experiments of *mig-17* pharynx and gonadal defects. *mig-17(k174)* young adult hermaphrodites having extrachromosomal arrays *Ex[mig-17*::*GFP]*, *Ex[mig-17(E303Q)*::*GFP]*, *Ex[mig-17(ΔDI)*::*GFP]*, and *Ex[mig-17(ΔGly1-9)*::*GFP]* (Ihara and Nishiwaki 2007) were analyzed. (A) Pharynx length. Data are shown as the mean ± SD (*n* = 30). (B) Gonad abnormality (*n* = 30 for WT, *n* = 60 for others). (C) Body length. Data are shown as the mean ± SD (*n* = 30). *P*-values for Fisher’s exact test against WT (black) and *mig-17(k174)* (magenta) are indicated: ** *P* < 0.01, *P < 0.05.

To explore the function of individual domains of MIG-17, as well as its glycosylation, in the regulation of pharynx length and DTC migration, we conducted transgenic rescue experiments of *mig-17(k174)* using extrachromosomal transgenic arrays (Ihara and Nishiwaki 2007). The transgenic array containing the wild-type *mig-17*::*GFP* fusion construct *Ex[mig-17*::*GFP]* fully rescued both pharyngeal and gonadal phenotypes (Figure 5, A–C). The transgenic arrays *Ex[mig-17(E303Q)*::*GFP]*, which lacks catalytic activity, *Ex[mig-17(ΔDI)*::*GFP]*, which lacks the DI domain, and *Ex[mig-17(ΔGly1-9)*::*GFP]*, which lacks all nine potential N-glycosylation sites, failed to rescue the pharyngeal phenotype. Similar results were obtained for the posterior gonadal defects, although these mutant constructs significantly rescued the anterior gonadal defects (Figure 5B). These results indicate that the catalytic activity, the DI domain, and N-glycan modifications are essential for regulating pharynx length.

### MIG-17-Venus localizes to the pharyngeal basement membrane

**Figure 6:**
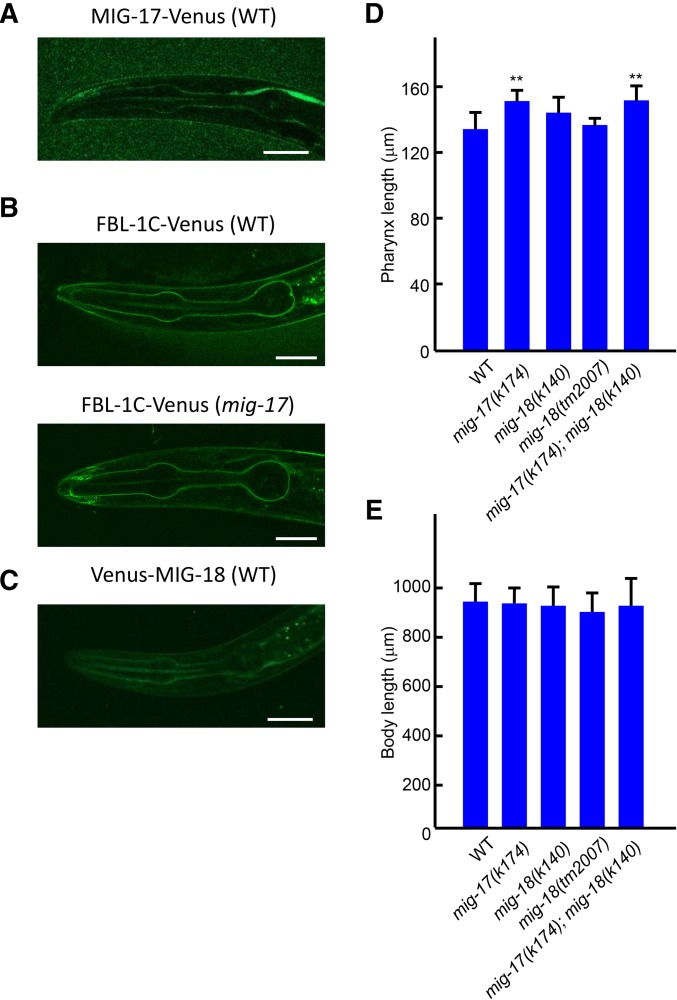
Localization of MIG-17, FBL-1C and MIG-18, and effects of *mig-18* mutations on pharynx length. (A–C) Confocal images of L3 larvae expressing MIG-17-Venus in WT (A), FBL-1C-Venus in WT (upper), and *mig-17(k174)* mutant (lower) (B), and Venus-MIG-18 in WT (C) backgrounds. Bar: 20 μm. (D), (E) Effects of *mig-18* mutations on pharynx length. Pharynx length (D) and body length (E) of young adult hermaphrodites were analyzed. Data are shown as the mean ± SD (*n* = 20). *P*-values for Fisher’s exact test against WT are indicated: ** *P* < 0.01.

We examined expression of the MIG-17-Venus functional fusion. MIG-17-Venus was observed to localize to the pharyngeal basement membrane (Figure 6A) as well as to the gonadal basement membrane (Kim *et al.* 2014). The pharyngeal localization of MIG-17-Venus was first detected in the twofold stage embryo and continued to the adult stage. The localization was strongest in L3 larvae, and became weaker in late larval and adult animals (data not shown).

MIG-17 activity is required for efficient accumulation of FBL-1C/fibulin-1C in the gonadal basement membrane, which is important for directional migration of gonadal DTCs (Kubota *et al.* 2004; Kim *et al.* 2014). We examined whether FBL-1C also localizes to the pharyngeal basement membrane, and whether the localization is affected by *mig-17* mutations. We found that FBL-1C-Venus, a functional fusion (Kubota *et al.* 2004), localized to the pharyngeal basement membrane, but its accumulation was not reduced in the *mig-17(k174)* mutant background compared with the wild type (Figure 6B, and Supplemental Material, Figure S1), suggesting that, in contrast to the case in the gonadal basement membrane, MIG-17 is not required for the recruitment of FBL-1C to the pharyngeal basement membrane.

### MIG-18, a cofactor of MIG-17, is not required for the control of pharynx length

MIG-18 is a novel secreted protein whose loss-of-function mutations result in DTC migration defects similar to those in the *mig-17* mutations. Based on the genetic evidence, we proposed that it acts as a cofactor of MIG-17 to regulate DTC migration (Kim *et al.* 2014). We examined MIG-18 expression using Venus-MIG-18, a functional fusion protein (Kim *et al.* 2014). We observed that Venus-MIG-18 localizes to the pharyngeal basement membrane from the twofold embryonic stage through the adult stage, and that the basement membrane accumulation was strongest during the L3 stage (Figure 6C, and data not shown). Thus, MIG-18 localized to the pharynx similar to MIG-17. Mutants with the putative null alleles, *mig-18(k140)* and *mig-18(tm2007)*, had pharynges of a similar length to those in wild-type animals. The length of the pharynx in *mig-17(k174)*; *mig-18(k140)* double mutants was elongated and comparable to that of the *mig-17(k174)* single mutants (Figure 6, D and E). These results suggest that MIG-18 is not required for the regulation of pharynx length, although it has a substantial function in DTC migration (Kim *et al.* 2014).

### Mutations in type IV collagen and fibulin-1 suppress the pharyngeal phenotype of mig-17

**Figure 7:**
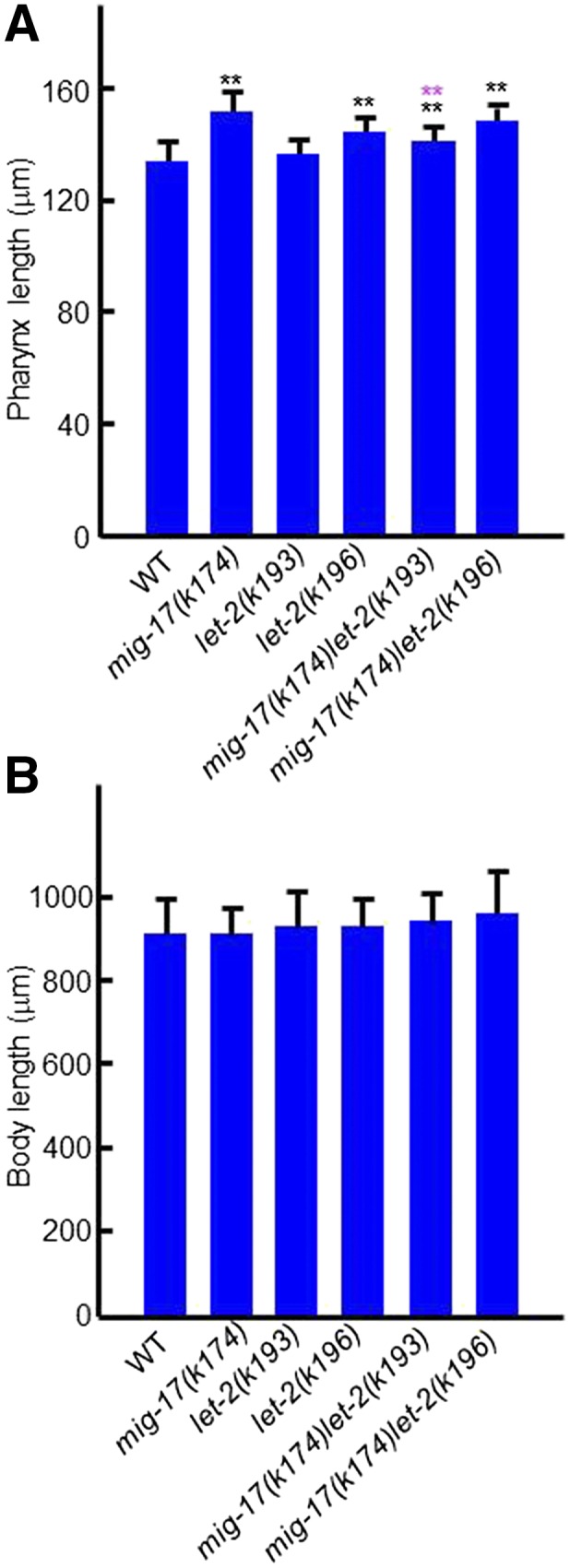
Effects of *let-2* mutations on pharynx length. (A), (B) Pharynx length (A) and body length (B) of young adult hermaphrodites were analyzed. Data are shown as the mean ± SD (*n* = 20). *P*-values for Fisher’s exact test against WT (black) and *mig-17(k174)* (magenta) are indicated: ** *P* < 0.01.

**Figure 8:**
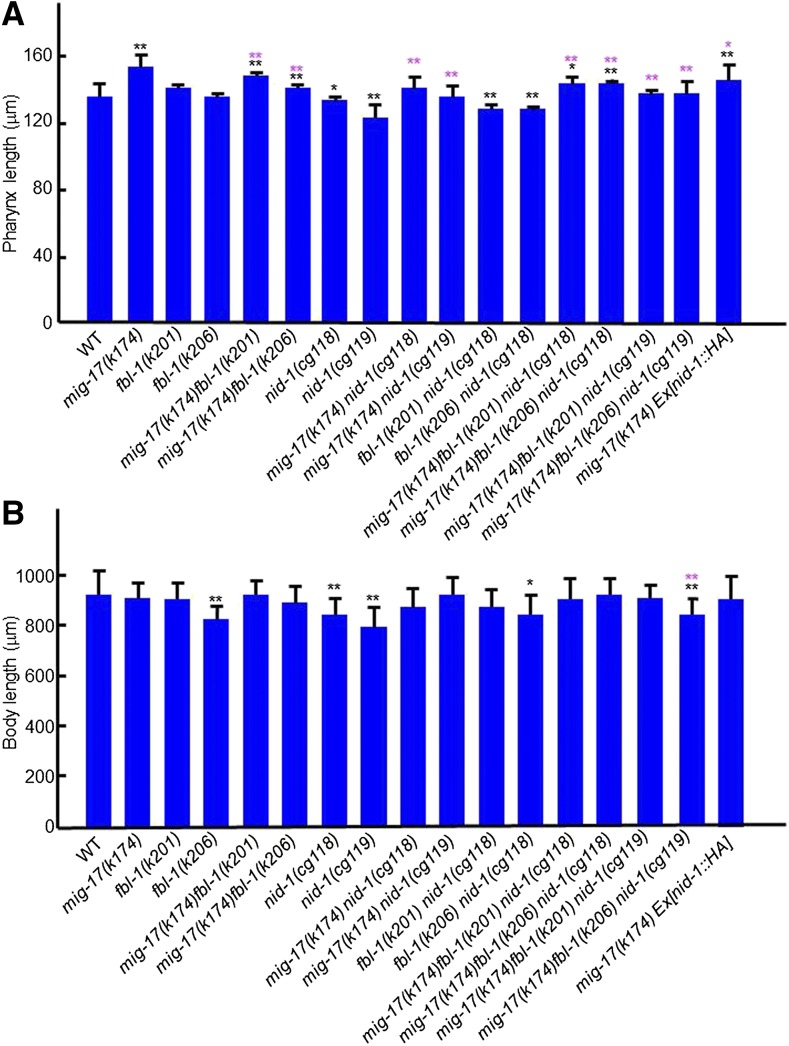
Effects of *fbl-1* and *nid-1* mutations on pharynx length. (A), (B) Pharynx length (A) and body length (B) of young adult hermaphrodites were analyzed. Data are shown as the mean ± SD (*n* = 20). *P*-values for Fisher’s exact test against WT (black) and *mig-17(k174)* (magenta) are indicated: ** *P* < 0.01, * *P* < 0.05.

The gonadal DTC migration defects of *mig-17* mutants can be suppressed by the gain-of-function mutations caused by amino acid substitutions in *let-2* (encoding the α2 subunit of type IV collagen) and *fbl-1* (encoding fibulin-1) (Kubota *et al.* 2004, 2008). When *mig-17(k174)* was combined with suppressor *let-2* mutations *k193* or *k196*, *k193* suppressed the pharyngeal phenotype, whereas *k196* did not (Figure 7, A and B). When *mig-17(k174)* was combined with suppressor *fbl-1* mutations *k201* or *k206*, they both suppressed the pharyngeal defects (Figure 8, A and B). Because the suppression of gonadal defects of *mig-17* by *fbl-1(gf)* mutations depends on *nid-1*, which encodes nidogen in *C. elegans* (Kubota *et al.* 2008), we asked whether this is also the case in pharyngeal defects. First, we combined the *nid-1(cg118)* (partial loss of function), or *nid-1(cg119)* (complete loss of function), allele with *mig-17(k174)*, and observed that they each suppressed the pharyngeal phenotype of *mig-17* (Figure 8, A and B). This was unexpected because *nid-1(cg119)* enhances *mig-17(k174)* with respect to gonadal defects (Kubota *et al.* 2008). When *nid-1* mutants were then combined with *mig-17(k174)*; *fbl-1(k201)* or *mig-17*; *fbl-1(k206)*, the *mig-17* pharynx length defect was still suppressed. Overexpression of NID-1-hemagglutinin (HA) partially suppresses the DTC migration defects of *mig-17* mutants (Kubota *et al.* 2008). We examined pharynx length in a *mig-17(k174)* mutant that overexpresses NID-1-HA (Figure 8, A and B) and observed partial suppression in the transgenic line. Therefore, either a reduction or increase in the NID-1 dosage appears to suppress the *mig-17* pharynx phenotype. Thus, it is likely that, although *let-2*, *fbl-1* and *nid-1* act to control the length of the pharynx in the MIG-17 pathway, their roles are not always the same as their roles in the control of gonadal DTC migration.

### Glycosylation mutants having mig-17-like gonadal defects exhibit short body length phenotypes

**Figure 9:**
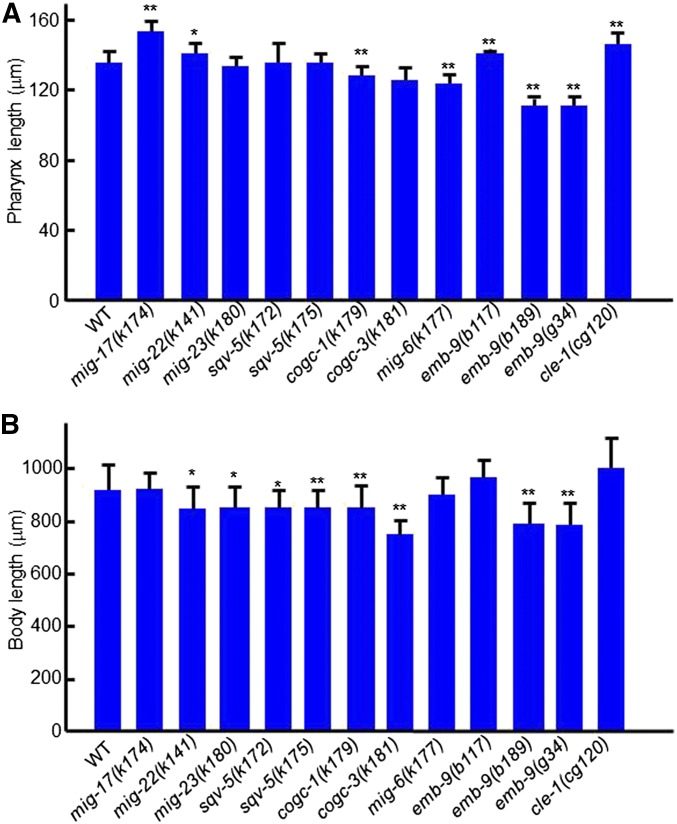
Effects of mutations affecting glycosylation and basement membrane components on pharynx length. (A), (B) Pharynx length (A) and body length (B) of young adult hermaphrodites were analyzed. Data are shown as the mean ± SD (*n* = 20). *P*-values for Fisher’s exact test against WT are indicated: ** *P* < 0.01, * *P* < 0.05.

Mutations in genes that act in protein glycosylation in the Golgi apparatus cause gonadal DTC migration-defective phenotypes similar to those observed in the *mig-17* mutants. These include *sqv-5* (chondroitin synthase), *mig-22* (chondroitin polymerizing factor), *mig-23* (Golgi NDPase), and *cogc-1* and *cogc-3* (components of conserved oligomeric Golgi complex) (Nishiwaki *et al.* 2004; Kubota *et al.* 2006; Suzuki *et al.* 2006). We examined mutant alleles of these genes, and found that each of these mutations resulted in a shorter body length relative to the wild type, and that a slight elongation of the pharynx was present only in *mig-22(k141)* animals (Figure 9, A and B). Because of the shorter body length, it was not clear whether the other mutants affected pharynx length. Although *mig-23*, *cogc-1*, and *cogc-3* mutants partially compromise the function of MIG-17 in DTC migration through their misglycosylation (Nishiwaki *et al.* 2004; Kubota *et al.* 2006), it may still function adequately to control pharynx length.

### Mutations in basement membrane proteins affect the length of the pharynx and body

*mig-6* encodes the basement membrane protein papilin. The class-s alleles of *mig-6*, which affect its short splicing isoform, exhibit intergenic noncomplementation with *mig-17* mutants with respect to the DTC migration phenotype, and therefore *mig-6* is proposed to act in the same genetic pathway as *mig-17* to control DTC migration (Kawano *et al.* 2009). The *mig-6(k177)* class-s mutants, however, showed a rather shortened pharynx (Figure 9, A and B), suggesting that *mig-6* may not function with *mig-17* to control the length of the pharynx. *emb-9* encodes the α1 subunit of type IV collagen, which forms a collagen trimer with the α2 subunit/LET-2. Animals carrying one of three *emb-9* mutations, which are embryonic or larval lethal at 25°, were reared at 20°, at which temperature they remain viable. The *b117* allele produced a slightly elongated pharynx, whereas the *b189* and *g34* alleles had shorter pharynges and bodies compared with the wild type. We also examined an allele of *cle-1*, which encodes basement membrane type XVIII collagen (Ackley *et al.* 2001) and observed a significant increase in the length of the pharynx, as was noted in the *emb-9(b177)* mutants (Figure 9, A and B). These results indicate that mutations in basement membrane collagens affect the control of the pharynx and body length.

## Discussion

### MIG-17 activity is involved in pharynx length regulation

In this study, we showed that an ADAMTS family metalloprotease, MIG-17, which is required for directional migration of gonadal DTCs, also acts in the regulation of pharynx length. We found that the pharynx of *mig-17* mutants becomes longer compared with the wild type after the L2 stage. Consistent with this observation, MIG-17-Venus strongly accumulated in the pharyngeal basement membrane during the L3 stage. These results suggest that MIG-17 negatively regulates pharynx elongation from the basal side of the pharynx. By expressing various mutant constructs in the *mig-17(k174)* null mutant, we found that the catalytic activity, the C-terminal DI domain and N-glycosylation of MIG-17 are essential for the regulation of pharynx length as in the case of the control of DTC migration. We previously showed that the MIG-17 pro-enzyme is converted into the mature enzyme by autoproteolytic removal of its prodomain (Ihara and Nishiwaki 2007). The catalytic activity of mature MIG-17 is important for controlling DTC migration, especially for migration of the posterior DTCs. Also, the DI domain is essential for prodomain processing (Ihara and Nishiwaki 2007). Thus it is likely that MIG-17 can be regulated in a similar manner in the pharyngeal basement membrane.

### MIG-17 acts through different molecular mechanisms in pharynx and gonad development

An analysis of molecules involved in the MIG-17 pathway that regulates DTC migration revealed distinct aspects of these molecules in the regulation of pharynx length. MIG-18, a cofactor of MIG-17 in gonadal DTC migration, was not required for pharynx length regulation. FBL-1C, which accumulates in the gonadal basement membrane in a strongly MIG-17-dependent manner, accumulated normally in the pharyngeal basement membrane in the absence of MIG-17.

Although both *let-2* alleles, *k193* and *k196*, strongly suppress DTC migration defects (Kubota *et al.* 2008), only *k193* suppressed the pharynx phenotype of *mig-17*. *k193* and *k196* are amino acid substitutions in the C-terminal noncollagenous (NC1) domain and the triple helical domain of type IV collagen, respectively. The NC1 domain is important for the linkage of NC1 domains of two type IV collagen trimers (Yurchenco and Schittny 1990; Sundaramoorthy *et al.* 2002). Thus, the type IV collagen meshwork that is formed in *k193* mutants could be partially disorganized because of the aberrant formation of intermolecular bonds between NC1 domains. In contrast, *k196* could affect folding or stability of part of the triple-helical structure, which may also result in disorganization of the meshwork. We speculate that the suppressor *let-2* mutations could mimic the type IV collagen remodeling in the basement membrane that is normally elicited by MIG-17. Therefore, the different suppression outcomes by *k196* in the formation of the pharynx and the gonad could reflect the different actions of MIG-17 in these basement membrane regions.

The *nid-1* mutations suppressed the *mig-17* pharyngeal phenotype, in contrast to its enhancement of *mig-17* gonadal defects. *nid-1* appears to act antagonistically to *mig-17* in the regulation of pharynx length. The observation that *nid-1* mutations themselves lead to shorter pharynges compared with wild type supports this idea (Figure 8A). NID-1 strongly localizes to the basement membrane of migrating DTCs but weakly to the pharyngeal basement membrane (Kang and Kramer 2000). In addition, *nid-1* produces three different splicing isoforms, NID-1A, B, and C (Kang and Kramer 2000). Thus, it might be possible that qualitative and/or quantitative differences in NID-1 expression may be the cause for distinct activities of NID-1 in the MIG-17–dependent mechanisms in the pharynx and the gonad.

### Basement membrane collagens are involved in pharynx length regulation

Basement membrane type IV collagen plays important roles in tissue morphogenesis (Morrissey and Sherwood 2015). We also showed in this study that mutations in type IV collagen affect pharynx length. Four of the five type IV collagen mutants examined—*let-2(k196)* and *emb-9(b117*, *b189*, and *g34)*—consist of substitutions of the glycines in the tripeptide repeats (Gly-X-Y) in the triple helical domain. Interestingly, *let-2(k196)* and *emb-9(b117)* showed an elongated pharynx, whereas *emb-9(b189)* and *emb-9(g34)* had a shortened pharynx. The type IV collagen molecule is a triple-helical trimer consisting of two α1/EMB-9 subunits and one α2/LET-2 subunit. The triple helix is formed by successive Gly-X-Y repeats, where X is often proline and Y is often lysine (Kramer 2005). Both EMB-9 and LET-2 molecules have intrinsic interruptions in the Gly-X-Y sequence, which are more often found in the N-terminal half of the triple-helical domain, and therefore the thermal stability of these molecules is higher in the C-terminal than the N-terminal region (Sibley *et al.* 1994). The lethal phenotypes of glycine substitution mutations in *emb-9* are stronger when they occur in the N-terminal half (*b189* and *g34*) than in the C-terminal half (*b117*) (Gupta *et al.* 1997). The *let-2(k196)* mutation is in the C-terminal half (Kubota *et al.* 2008). Therefore it might be possible that glycine substitutions in the C-terminal high-stability region result in pharynx elongation, whereas those in the N-terminal low-stability region result in pharyngeal shortening. We speculate that MIG-17 may modulate the structure or conformation of the C-terminal half of the triple-helical domain to achieve a pharynx of the proper length.

We found that *cle-1(cg120)* results in pharynx elongation similar to that in the *emb-9(b117)* and *let-2(k196)* mutants. CLE-1/type XVIII collagen is widely distributed in the basement membrane, and *cle-1(cg120)* is a deletion mutant lacking the NC1 domain, which contains the endostatin (an angiogenesis inhibitor in mammals) domain (Ackley *et al.* 2001). Because *cle-1(cg120)* mutants show DTC migration defects (that phenotypically differ from those of *mig-17*) (Ackley *et al.* 2001), endostatin is suggested to have a role in this process. Thus, it might be possible that endostatin also acts in pharynx length control.

### Pharynx elongation in mig-17 mutants

Our finding indicates that the pharynx of *mig-17* mutants is elongated because of the elongation of cells rather than an increase in the cell number. The pharynx is ∼60 μm long when worms hatch, and it elongates to ∼130 μm long by the adult stage (about a twofold increase). During the same period, the body size grows from ∼200 to 900 μm (an ∼4.5-fold increase). Thus, the rate of pharyngeal elongation is much lower than that of body elongation. Recently, it was shown that the embryonic pharynx behaves like a spring in which the anterior end is attached to the mouth, and the posterior end attached to the surrounding epidermis (Kelley *et al.* 2015). During embryonic 1.5- and 3-fold stages, the pharyngeal spring is stretched with increasing tensile force along the anterior-posterior axis because of the elongation of the epidermis. If this is also the case in the larval pharynx, the active elongation of the larval body, or the elongation of the epidermis, should stretch the pharynx during its elongation. Because epidermal elongation is much greater than pharyngeal elongation, we should postulate some slippage between the pharynx end and the epidermis. Because the pharyngeal end interacts with the surrounding epidermis through their basement membranes, MIG-17 may normally act to weaken the friction or adhesiveness between these basement membranes to produce a pharynx of proper length. Loss of MIG-17 activity may enhance the adhesion and therefore confer a greater stretching force, resulting in pharynx elongation. However, it is also possible that the larval pharynx expands actively rather than passively by stretching. In this case, MIG-17 may act to stiffen the pharyngeal basement membrane to inhibit its overgrowth. Interestingly, type IV collagen functions in organ morphogenesis through providing a constricting force in *Drosophila* (Haigo and Bilder 2011; Pastor-Pareja and Xu 2011). MIG-17 may affect the stiffness of the pharyngeal basement membrane similarly by modulating the type IV collagen meshwork. Further studies are needed to distinguish between these possibilities.

### ADAMTS function controlling organ shape can be evolutionarily conserved

Our finding demonstrates that MIG-17/ADAMTS functions in organ shape control through its remodeling activity in the basement membrane in *C. elegans*. Another ADAMTS in *C. elegans*, ADT-2, regulates body size by modulating TGFβ signaling and cuticle collagen organization (Fernando *et al.* 2011). This function in shape control appears to be conserved in mammalian ADAMTS proteins, which also act in ECM remodeling. Mutations in human ADAMTS10 and ADAMTS17 cause Weill-Marchesani syndrome, which is characterized by short stature and eye abnormalities including microspherophakia (the lens of the eye is smaller than normal and spherically shaped) (Dagoneau *et al.* 2004; Morales *et al.* 2009; Shah *et al.* 2014). It is worth noting that MIG-17, which acts in pharynx length regulation in *C. elegans*, has a structural similarity to ADAMTS10 and 17 in that they all share C-terminal protease and lacunin (PLAC) domains (Ihara and Nishiwaki 2007). Further analysis of *C. elegans* ADAMTS proteinases would shed light on the detailed molecular mechanisms and the consequent physical interactions between the ECM and the cells that finally determine organ shape.

## Supplementary Material

Supplemental Material
