# Supplementary material for: Organ Length Control by an ADAMTS Extracellular Protease in Caenorhabditis elegans
Source: G3 (Bethesda). 2016 Mar 17;6(5):1449–57. doi: 10.1534/g3.116.028019 (PMC4856095; doi:10.1534/g3.116.028019)
Supplement: Supplemental Material [file supp_6_5_1449__index.html]

Organ Length Control by an ADAMTS Extracellular Protease in Caenorhabditis elegans — Supplemental Material 

# Organ Length Control by an ADAMTS Extracellular Protease in *Caenorhabditis elegans*

## Supplemental Material for Shibata *et al.*, 2016

**Files in this Data Supplement:**

- Figure S1 - Quantification of FBL-1C-Venus. (.jpg, 147 KB)
