# Supplementary figures and images for: Organ Length Control by an ADAMTS Extracellular Protease in Caenorhabditis elegans
Source: G3 (Bethesda). 2016 Mar 17;6(5):1449–57. doi: 10.1534/g3.116.028019 (PMC4856095; doi:10.1534/g3.116.028019)

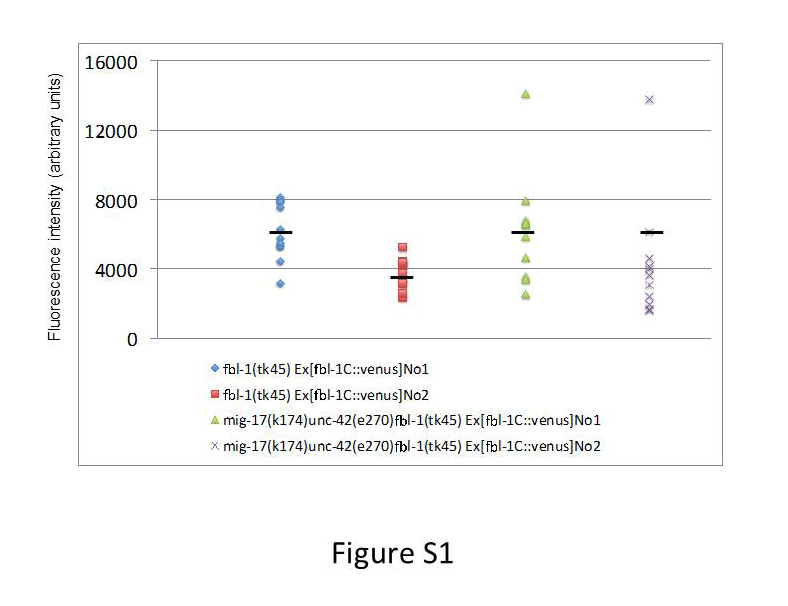

Supplement: Supplemental Material [file supp_g3.116.028019_FigureS1.jpg]
